# Effects of Trehalose, Sodium Hyaluronate and N-Acetyl Aspartyl-Glutamic Acid Artificial Tears on Ocular Surface Parameters in Glaucoma Patients Receiving Preserved and Preservative-Free Prostaglandins

**DOI:** 10.3390/jcm15083012

**Published:** 2026-04-15

**Authors:** Alessio Martucci, Flavia Quaranta Leoni, Noemi Valentini, Roberto Pietro Sorge, Raffaele Mancino, Francesco Aiello, Massimo Cesareo, Carlo Nucci

**Affiliations:** 1 Ophthalmology Unit, Department of Experimental Medicine, University of Rome “Tor Vergata”, Via Montpellier 1, 00133 Rome, Italy; flavia.quarantaleoni@ptvonline.it (F.Q.L.); noemi04valentini@gmail.com (N.V.); mancino@med.uniroma2.it (R.M.); francesco.aiello@ptvonline.it (F.A.); massimo.cesareo@ptvonline.it (M.C.); nucci@med.uniroma2.it (C.N.); 2 Laboratory of Biometry, Department of Systems Medicine, University of Rome “Tor Vergata”, 00133 Rome, Italy; sorge@uniroma2.it

**Keywords:** primary open-angle glaucoma (POAG), prostaglandin analogue, N-acetyl-aspartyl-glutamate (NAAGA), trehalose, sodium hyaluronate, ocular surface, artificial tears

## Abstract

**Background:** To evaluate the effects of a preservative-free artificial tear formulation containing trehalose, sodium hyaluronate, and N-acetyl-aspartyl-glutamate (NAAGA) on ocular surface parameters and quality of life in patients with primary open-angle glaucoma (POAG) treated with preserved versus preservative-free prostaglandin analogues. **Methods:** For this prospective, observational clinical study, thirty-eight patients (76 eyes) with POAG receiving stable topical prostaglandin therapy were enrolled and divided into two groups: preserved prostaglandins (Group 1, n = 44) and preservative-free prostaglandins (Group 2, n = 32). All patients received adjunctive preservative-free artificial tears (trehalose, sodium hyaluronate, NAAGA) three times daily for one month. Assessments at baseline (T0) and 1 month (T1) included best-corrected visual acuity (BCVA), intraocular pressure (IOP), contrast sensitivity, Schirmer test, tear break-up time (BUT), Efron grading scale, Ocular Surface Disease Index (OSDI), visual field (VF) indices (Mean Deviation (MD), Pattern Standard Deviation (PSD), Visual Field Index (VFI)), and quality of life (QoL) measured using Visual Analogue Scales (VAS). **Results:** After 1 month, both groups demonstrated significant improvement in ocular surface parameters. Schirmer test increased by approximately 4–5 mm (*p* = 0.001 in both groups), and BUT improved by 5 s (*p* = 0.001 in both groups). OSDI scores significantly decreased (Group 1: –18.5; Group 2: –23; *p* = 0.001 for both), and Efron grading significantly improved (*p* = 0.001 in both groups). Artificial tears-related QoL markedly increased in both groups (*p* = 0.001), while pathology-related QoL remained unchanged. IOP showed a modest but significant reduction in both groups (Group 1 *p* = 0.011; Group 2 *p* = 0.003), without intergroup differences. VFI significantly improved in both groups from T0 to T1 (Group 1 *p* = 0.013; Group 2 *p* = 0.04). Group 1 also showed an improvement in terms of PSD (*p* = 0.025). **Conclusions:** Adjunctive treatment with preservative-free artificial tears containing trehalose, sodium hyaluronate, and NAAGA significantly improved tear film stability VF indexes, ocular surface signs and symptoms, and patient-reported QoL in POAG patients treated with prostaglandins, regardless of preservative status. Routine ocular surface optimization should be considered an integral component of comprehensive glaucoma management.

## 1. Introduction

Glaucoma is a chronic, progressive optic neuropathy that requires long-term topical therapy to control intraocular pressure and prevent disease progression. While topical treatment remains the mainstay of glaucoma management, increasing evidence has highlighted its detrimental effects on the ocular surface. Multiple observational studies and reviews have consistently reported a high prevalence of Ocular Surface Disease (OSD) in patients with glaucoma, particularly among those receiving chronic topical therapy, with prevalence rates exceeding 50% in several cohorts [1,2,3]. OSD in glaucomatous patients is frequently underrecognized and may be present even in the early stages of treatment, often independently of glaucoma severity [1,4,5].

The pathogenesis of ocular surface alterations in glaucoma patients is multifactorial [4]. Chronic exposure to topical antiglaucoma medications has been associated with tear film instability, epithelial damage, conjunctival inflammation, and meibomian gland dysfunction [4,6]. These changes have been linked both to the active compounds and, more prominently, to preservatives such as benzalkonium chloride, which induce cumulative toxic and pro-inflammatory effects on the corneal and conjunctival epithelium [7,8]. Several studies have demonstrated a direct relationship between the number of topical medications, duration of treatment, and severity of ocular surface signs and symptoms [3,5].

Among antiglaucoma medications, prostaglandin analogues are widely used as first-line therapy and have been specifically implicated in ocular surface impairment [9]. Clinical studies have shown that patients treated with prostaglandin analogues frequently exhibit reduced tear film break-up time, increased corneal and conjunctival staining, and higher symptom scores related to dry eye disease [5,10]. The ocular surface damage associated with prostaglandin therapy appears to be influenced by both the pharmacological effect of the molecules and the presence of preservatives, with long-term treatment leading to progressive surface alterations [6,10].

Importantly, Ocular Surface Disease in glaucoma patients has been shown to significantly affect patient-reported outcomes and quality of life [10,11]. Several investigations have demonstrated that symptoms related to dry eye disease, such as ocular discomfort, burning, foreign body sensation, and visual fluctuation, are major contributors to patient dissatisfaction with glaucoma treatment [9,12]. In some studies, the severity of ocular surface symptoms was more strongly associated with reduced quality of life (QoL) than traditional clinical parameters of glaucoma, including intraocular pressure control [11,13]. Furthermore, ocular surface discomfort has been identified as a key factor negatively influencing treatment adherence and persistence [9,12].

Given the chronic nature of glaucoma therapy, preserving ocular surface health has emerged as an essential component of comprehensive disease management [1,8]. Expert opinions and systematic reviews have emphasized the importance of early identification and treatment of OSD in glaucomatous patients, particularly in those receiving prostaglandin analogues [6,14]. Adjunctive therapies aimed at protecting the ocular surface, improving tear film stability, and reducing epithelial damage have been shown to mitigate treatment-related toxicity and improve patient comfort [8,9].

The primary objective of this study was to assess whether a standardized ocular surface regimen, consisting of preservative-free artificial tears containing trehalose (3%), sodium hyaluronate (0.15%), and N-acetyl-aspartylglutamic acid (NAAGA 2.45%), could mitigate the deleterious effects of chronic topical glaucoma therapy on the ocular surface.

Specifically, the study aimed to determine whether adjunctive tear supplementation may offset the additional adverse impact associated with preservative-containing prostaglandin analogues, while simultaneously improving ocular surface parameters in patients treated with preservative-free formulations.

By administering the same supportive therapy to both groups, the study was designed to evaluate whether treatment-related ocular surface impairment in glaucoma patients could be effectively counterbalanced in these scenarios in the short term (1 month) thanks to the mild anti-inflammatory action of the components.

Lubricants containing sodium hyaluronate have demonstrated efficacy in reducing ocular surface damage and improving clinical signs and symptoms of dry eye disease [15,16], including in patients treated with preserved antiglaucoma medications [16]. These findings support the use of targeted lubricating therapies as a complementary strategy in glaucoma patients with concomitant Ocular Surface Disease.

## 2. Material and Methods

This observational clinical study was carried out between December 2024 and December 2025 and enrolled 2 groups of patients with a confirmed diagnosis of primary open-angle glaucoma (POAG) who were undergoing stable topical hypotensive therapy with preserved (Group 1) or preservative-free (Group 2) prostaglandin analogue and reported symptoms consistent with ocular surface discomfort. The study was performed in compliance with the Declaration of Helsinki, Good Clinical Practices, International Organization for Standardization 14155, and local regulations. The study was approved by an independent ethics committee (Comitato Etico Territoriale Lazio Area 2) registration number 293.24c3T2ptv (30 November 2024), and all patients gave written informed consent.

Inclusion criteria comprised age over 18 years, reliable visual field (VF) testing, and stable intraocular pressure (IOP) under therapy for at least three months.

Exclusion criteria included previous ocular surgery within six months, active ocular infection or inflammation unrelated to glaucoma therapy, contact lens wear, and systemic diseases known to significantly affect the ocular surface.

Patients were evaluated at baseline (T0) and after one month of regular use of preservative-free ophthalmic solution containing trehalose (3%), sodium hyaluronate (0.15%) and N-acetyl aspartylglutamic acid (NAAGA 2.45%) (T1), administered according to a standardized regimen (3 times daily in both eyes) in addition to their ongoing antiglaucoma treatment.

At both T0 and T1 visits, a comprehensive ophthalmological examination was performed. Best-corrected visual acuity (BCVA) was measured under standardized photopic lighting conditions at standard testing distance. Participants were instructed to read progressively smaller optotypes, and best-corrected visual acuity (BCVA) was recorded in decimal units. The test was performed monocularly with appropriate refractive correction in place, and the smallest line read with no more than one error was documented as the final visual acuity score. The device (CSO Vision Chart, Firenze, Italy) is equipped with a 19-inch (diagonal) high-definition Liquid Crystal Display screen (1280 × 1024 resolution), featuring a contrast ratio of 500:1 and a maximum luminance of 280 cd/m^2^. It includes a 50-button infrared remote control, allowing the operator to access all available tests remotely through simple and intuitive steps.

IOP was assessed using Goldmann applanation tonometry (Haag-Streit), considered the gold standard for IOP measurement. Values were expressed in mmHg.

Ocular surface symptoms were assessed using the Ocular Surface Disease Index (OSDI), a validated 12-item questionnaire designed to quantify the frequency of dry eye symptoms, visual disturbances, and environmental triggers over the previous week. Scores range from 0 to 100, with higher scores indicating greater symptom severity.

In addition, slit-lamp biomicroscopy was performed to grade ocular surface alterations using the Efron scale. The Efron grading scale is a standardized clinical grading system that classifies the severity of conjunctival hyperemia, corneal staining, and other surface signs on an ordinal scale, typically from 0 (normal) to 4 (severe).

Tear film stability and quality were further evaluated through multiple objective tests. Tear film break-up time (BUT) was measured after instillation of fluorescein dye; the interval between a complete blink and the first appearance of a dry spot on the corneal surface was recorded in seconds. Shorter break-up times indicate reduced tear film stability. The Schirmer test, performed without topical anesthesia, was used to quantify basal and reflex tear production. A standardized filter paper strip was placed in the lower fornix for five minutes, and the length of wetting (in millimetres) was recorded. Contrast sensitivity was assessed with the Pelli-Robson test using a standardized contrast sensitivity chart (CSO Vision Chart), which evaluates the ability to discern subtle differences in luminance between an object and its background, providing complementary information to high-contrast visual acuity testing. In the present study, data were expressed as percentages, with lower values indicating better performance (i.e., greater contrast sensitivity).

Functional assessment of glaucomatous damage was performed using automated perimetry, with a Humphrey Field Analyzer (Carl Zeiss Meditec, Inc., Dublin, OH, USA) employing a standard 24-2 testing algorithm. VF testing measures differential light sensitivity across the central 24 degrees of the VF and provides global indices such as Mean Deviation (MD), Pattern Standard Deviation (PSD), and Visual Field Index (VFI). VF tests were considered reliable if fixation losses were <20%, false-positive responses <15%, and false-negative responses <15%, in accordance with standard reliability criteria for automated perimetry [17]. Patient-reported outcomes regarding QoL were assessed using three specific questions administered at both time points. Responses were recorded on a Visual Analogue Scale (VAS) ranging from 1 to 100, where 1 indicated minimal impact and 100 indicated maximal impact. The first question (“How much does glaucoma therapy impact your quality of life?”) evaluated the perceived impact of antiglaucoma therapy on quality of life (therapy-related QoL). The second question (“How much does glaucoma impact your quality of life?”) assessed the perceived impact of glaucoma as a disease on overall QoL (pathology-related QoL). The third question (“How much has the use of tear substitutes improved your quality of life?”) investigated the perceived improvement in quality of life attributable to the use of artificial tears (artificial tears-related QoL). VAS scores were measured by asking patients to indicate a point along a 100 mm horizontal line corresponding to their subjective perception, which was then quantified numerically.

All assessments were conducted by the same experienced examiner to reduce inter-observer variability and were consistently performed at the same time of day, in the same examination room, and under identical lighting conditions.

AI-assisted tools (ChatGpt, 30 January 2025) were used to improve the language and readability of this manuscript. 

### Statistical Analysis

Data were collected in a standardized case report form and analyzed using appropriate statistical methods to compare pre- and post-treatment values, with significance set at *p* ≤ 0.05. Statistical analysis was performed using IBM SPSS Statistics (IBM SPSS Statistics for Windows, Vers. 25.0. Armonk, NY, USA: IBM Corp.). Statistical analysis was performed after assessing data distribution normality using the One-Sample Kolmogorov–Smirnov test. For normally distributed variables, paired Student’s *t*-tests were applied to compare pre- and post-treatment values. For non-normally distributed data, the Wilcoxon signed-rank test was used. A *p*-value ≤ 0.05 was considered statistically significant. The primary outcome measures were changes in ocular surface parameters and patient-reported QoL, while secondary outcomes included stability of intraocular pressure and VF indices.

## 3. Results

A total of 76 eyes of 38 patients were included in the analysis. Forty-four eyes were treated with preserved prostaglandins (Group 1) and 32 eyes with preservative-free prostaglandins (Group 2) for clinical parameters. Age and sex distribution did not differ significantly between groups (*p* = 0.070 and *p* = 0.933, respectively), confirming baseline demographic comparability. As most variables were not normally distributed according to Kolmogorov–Smirnov testing, data are presented as medians (min–max) and non-parametric analyses were applied.

**Figure 1:**
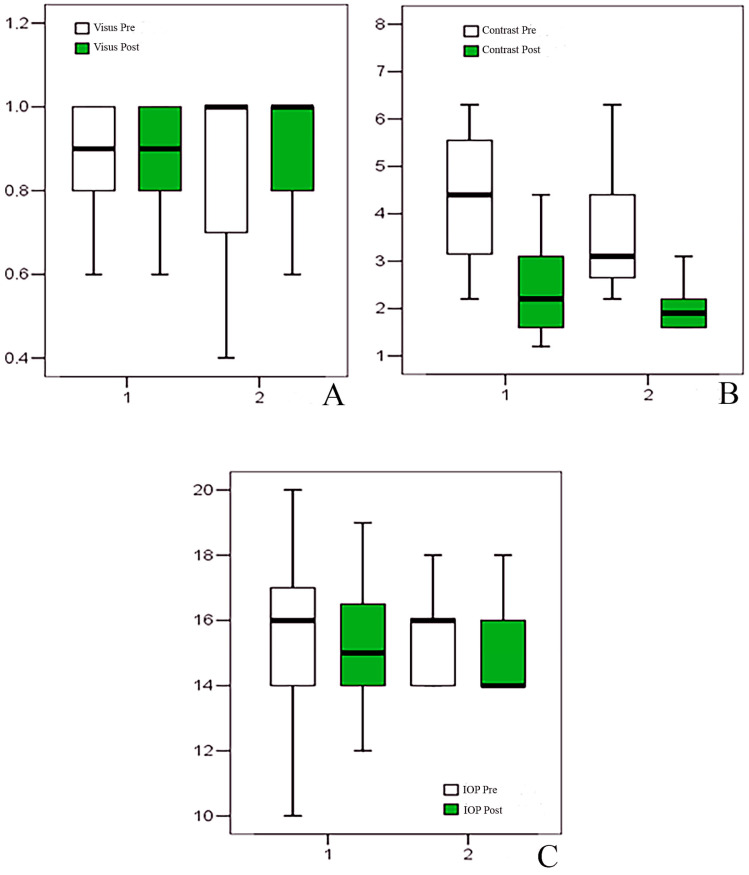
Box plot representing visus (**A**), contrast sensitivity test (**B**), and intraocular pressure (IOP) (**C**) results.

At T0, BCVA was homogeneous between groups (Group 1: 0.9 [0.1–1.0]; Group 2: 0.95 [0.1–1.0]; *p* = 0.466). Following one month of artificial tear treatment, BCVA showed a small but statistically significant improvement in both groups. In Group 1, median BCVA remained 0.9 but increased within the range (0.3–1.0), with a median change of 0.0 (0.0–0.2; *p* = 0.002). In Group 2, BCVA improved from 0.95 to 1.0 (0.3–1.0), with a median change of 0.0 (0.0–0.2; *p* = 0.001). The magnitude of change did not differ between groups (*p* = 0.377) (Table 1, Figure 1).

IOP was homogeneous between the two groups at T0 (Group 1: 16 [10,11,12,13,14,15,16,17,18,19,20]; Group 2: 16 [14,15,16,17,18]; *p* = 0.795). At T1, a modest but statistically significant reduction was observed in both groups. In Group 1, IOP decreased from 16 to 15 mmHg (10–19), with a median change of 0 (−3 to +2; *p* = 0.011). In Group 2, IOP decreased from 16 to 14 mmHg (14–18), with a median change of 0 (−2 to +1; *p* = 0.003). No significant intergroup difference was detected in IOP change (*p* = 0.251) (Table 1, Figure 1).

Contrast sensitivity differed at T0 between groups (Group 1: 4.4 [2.2–50]; Group 2: 3.1 [2.2–12.5]; *p* = 0.020). After treatment, contrast sensitivity significantly improved in both groups. Group 1 decreased from 4.4 to 2.2 (1.2–25), with a median change of −1.9 (−25 to −0.6; *p* = 0.001), while Group 2 decreased from 3.1 to 1.9 (1.6–4.4), with a median change of −1.5 (−8.1 to −0.6; *p* = 0.001). The change did not significantly differ between groups (*p* = 0.075) (Table 1, Figure 1).

**Figure 2:**
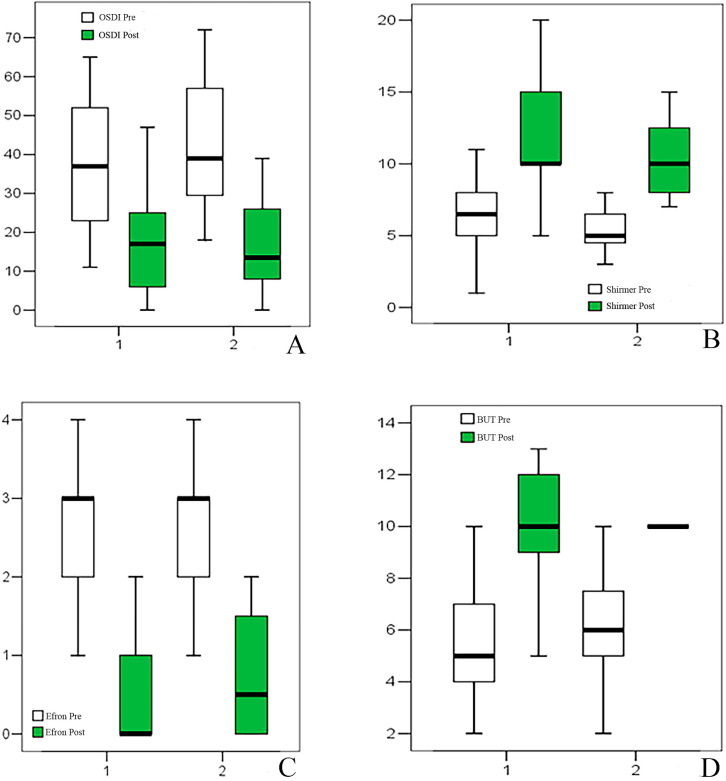
Box plot representing Ocular Surface Disease Index (OSDI) (**A**), Shirmer Test (**B**), Efron scale (**C**), and break-up time (BUT) (**D**) results.

Schirmer test values were comparable at T0 between groups (Group 1: 5 [2–15]; Group 2: 6 [2–12]; *p* = 0.057). Following treatment, a marked increase was observed in both groups. In Group 1, Schirmer values increased from 5 to 10 mm (4–20), with a median change of 4.5 (0–7; *p* = 0.001). In Group 2, values increased from 6 to 10 mm (6–15), with a median change of 4 (−2 to 6; *p* = 0.001). No significant intergroup difference in change was detected (*p* = 0.894) (Table 1, Figure 2).

Similarly, BUT was comparable between the two groups at T0 (Group 1: 6.5 [1–20]; Group 2: 5 [3–12]; *p* = 0.635). At T1, BUT increased significantly in both groups. In Group 1, it rose from 6.5 to 10 s (5–20), with a median increase of 5 s (0–10; *p* = 0.001). In Group 2, it increased from 5 to 10 s (7–20), with a median change of 5 s (2 to 10; *p* = 0.001). No significant difference in the magnitude of change was observed between groups (*p* = 0.118) (Table 1, Figure 2).

**Figure 3:**
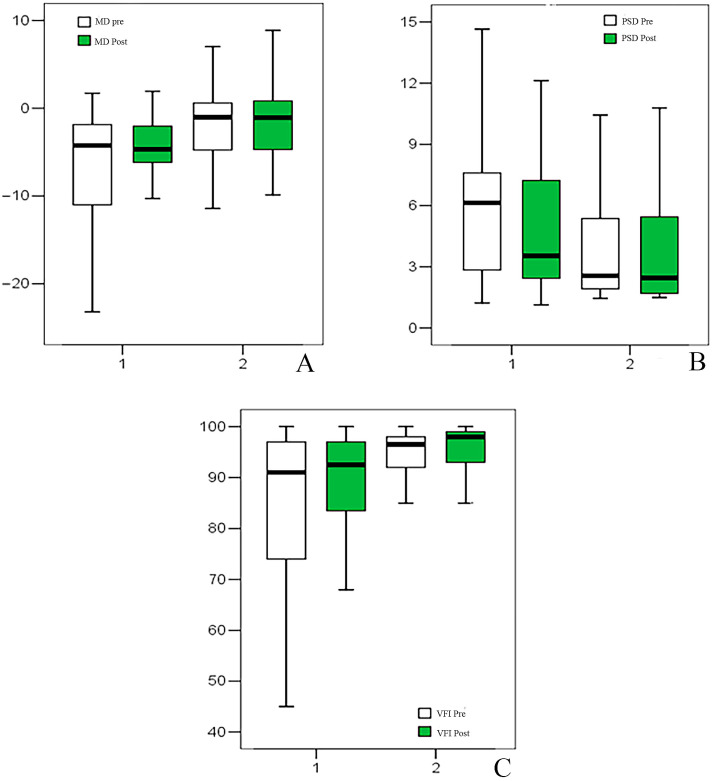
Box plot representing Mean Deviation (MD) (**A**), Pattern Standard Deviation (PSD) (**B**), Visual Field Index (**C**).

Regarding VF parameters, MD differed significantly between the two groups at T0 (Group 1: −4.25 [−23.22 to 1.72]; Group 2: −1.03 [−14.46 to 7.00]; *p* = 0.001). However, no significant pre-post changes were observed in either group (Group 1 *p* = 0.188; Group 2 *p* = 0.568), and the change did not differ between groups (*p* = 0.580). PSD also differed at baseline (Group 1: 6.13 [1.23–14.65]; Group 2: 2.56 [1.45–10.43]; *p* = 0.015). A modest reduction in PSD was observed only in Group 1 (median change −0.31 [−5.35 to 4.33]; *p* = 0.025), whereas no significant change occurred in Group 2 (*p* = 0.548); intergroup comparison of change was not significant (*p* = 0.337). VFI was lower in Group 1 at T0 (91 [28–100] vs. 96.5 [62–100]; *p* = 0.009). VFI increased slightly in both groups (Group 1: 91 to 92.5 [44–100], Δ = 1 [−16 to 47], *p* = 0.013; Group 2: 96.5 to 98 [78–100], Δ = 0 [−13 to 17], *p* = 0.040), with no significant difference in change between groups (*p* = 0.604) at T1 (Table 2, Figure 3).

OSDI showed no significant baseline differences between groups at T0. OSDI scores significantly decreased in both groups. In Group 1, OSDI decreased from 37 (11–65) to 17 (0–47), with a median reduction of −18.5 (−46 to −7; *p* = 0.001). In Group 2, it decreased from 39 (18–72) to 13.5 (0–39), with a median reduction of −23 (−57 to −6; *p* = 0.001). The magnitude of change did not differ significantly between groups (*p* = 0.234) (Table 3, Figure 2).

Efron scale showed no significant baseline differences between groups. Efron scale grading decreased significantly in both groups (Group 1: 3 to 0 [0–2], Δ = −2 [−4 to 0], *p* = 0.001; Group 2: 3 to 0.5 [0–2], Δ = −2 [−3 to 0], *p* = 0.001), without intergroup differences (*p* = 0.872) (Table 3, Figure 2).

**Figure 4:**
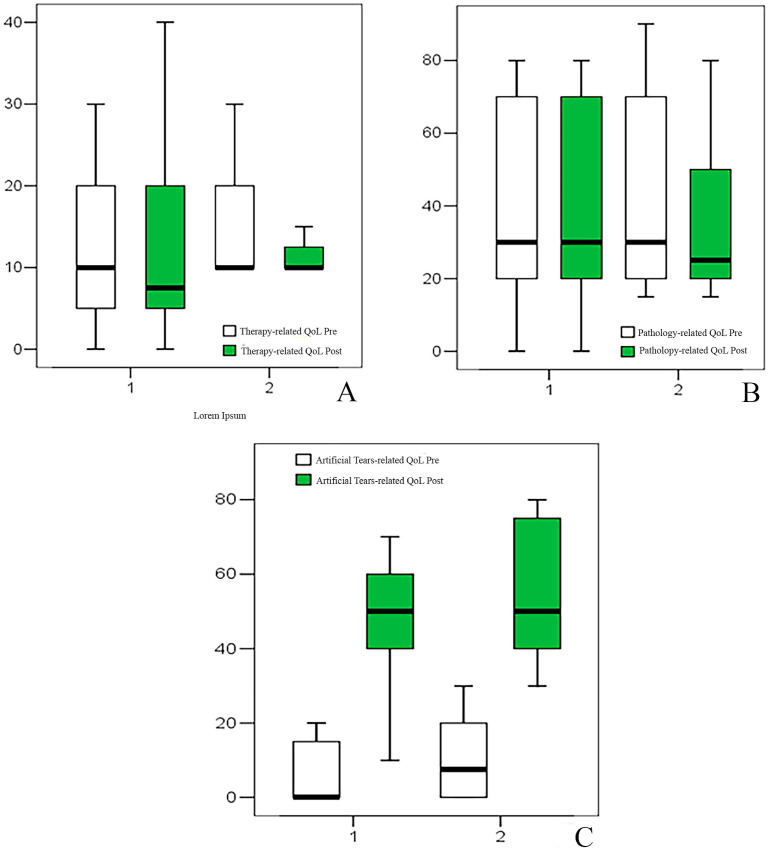
Box plot representing therapy- (**A**), pathology- (**B**), and artificial tears- (**C**) related quality of life (QoL) results.

Therapy-related QoL showed no significant baseline differences between groups. Therapy-related QoL improved modestly in Group 1 (10 to 7.5, Δ = 0 [−10 to 0], *p* = 0.038) and showed borderline improvement in Group 2 (10 to 10, *p* = 0.06), with no significant difference in change (*p* = 0.693) (Table 3, Figure 4).

Pathology-related QoL showed no significant baseline differences between groups. No significant pre-post variation was observed in pathology-related QoL in either group (Table 3, Figure 4).

Artificial tears-related QoL showed no significant baseline differences between groups. Conversely, artificial tears-related QoL showed a marked improvement in both groups in the post-treatment period. In Group 1, scores increased from 0 (0–20) to 50 (10–70), with a median change of 40 (10–70; *p* = 0.001). In Group 2, scores increased from 7.5 (0–30) to 50 (30–80), with a median change of 40 (10–80; *p* = 0.001), without significant intergroup difference at T1 (*p* = 0.589) (Table 3, Figure 4).

Overall, artificial tear treatment was associated with significant improvements in ocular surface parameters and patient-reported outcomes in both groups, while no consistent differential effect was observed between preserved and preservative-free prostaglandin users.

## 4. Discussion

OSD is increasingly recognized as a central component of the therapeutic burden in glaucoma management. The coexistence of glaucoma and dry eye has been described as a “dual dilemma,” in which chronic IOP-lowering therapy may compromise ocular surface integrity and negatively affect QoL [1,4]. While the prevalence and clinical impact of OSD in glaucoma patients are well documented [2,3,5,11,12,13], fewer studies have examined whether systematic adjunctive tear supplementation provides measurable benefits across different prostaglandin regimens. This study contributes to this gap by demonstrating that artificial tear therapy is associated with clinically meaningful improvements in both objective tear film parameters and patient-reported outcomes in patients treated with either preserved or preservative-free prostaglandins. For the purposes of this study, a preservative-free ophthalmic solution containing trehalose, sodium hyaluronate, and N-acetyl-aspartyl-glutamate (NAAGA) was administered in addition to the patients’ ongoing topical hypotensive therapy for glaucoma. The formulation was selected to address ocular surface alterations frequently associated with chronic antiglaucoma treatment, particularly in patients exposed to long-term topical medications. Artificial tears were instilled bilaterally according to a standardized regimen (e.g., one drop three times daily for the duration of the study), and no changes were made to the existing intraocular pressure-lowering therapy during the observation period.

Trehalose is a naturally occurring disaccharide found in numerous plants, microorganisms, and invertebrates capable of surviving extreme environmental conditions, particularly prolonged dehydration. Its biological relevance derives from the phenomenon of anhydrobiosis, defined as the ability of certain organisms to tolerate near-complete desiccation and subsequently recover normal function upon rehydration. At the cellular level, trehalose exerts protective, antioxidant, and membrane-stabilizing properties. It is believed to interact with phospholipid bilayers and proteins, preserving their structural integrity under osmotic and desiccating stress. In Ocular Surface Disease, trehalose has been shown to protect epithelial cells from hyperosmolar stress, reduce apoptosis, and stabilize the tear film, thereby improving both objective and subjective parameters of dry eye. Experimental and clinical evidence suggests that trehalose-containing formulations can reduce corneal staining and improve tear film stability in patients with ocular surface disorders [18,19,20,21,22,23,24,25].

Sodium hyaluronate (hyaluronic acid salt) is a well-known naturally occurring glycosaminoglycan present in various ocular tissues, including the vitreous body and the tear film. Due to its high molecular weight and viscoelastic properties, sodium hyaluronate has a remarkable capacity to bind and retain water molecules, thereby enhancing hydration and lubrication of the ocular surface. Its mucoadhesive characteristics allow prolonged residence time on the corneal and conjunctival epithelium, contributing to sustained symptomatic relief. Additionally, hyaluronic acid promotes epithelial wound healing by facilitating cell migration and proliferation. Numerous clinical studies have demonstrated the efficacy of sodium hyaluronate in improving tear film break-up time, reducing corneal staining, and alleviating dry eye symptoms [22,23]. In glaucoma patients, where chronic exposure to topical medications may disrupt tear film homeostasis and induce ocular surface inflammation, sodium hyaluronate provides both mechanical lubrication and biological support for epithelial recovery.

The combination of trehalose and sodium hyaluronate offers complementary and potentially synergistic effects. While trehalose primarily exerts cytoprotective and anti-desiccation activity at the cellular level, sodium hyaluronate enhances tear film stability and prolongs ocular surface hydration. Together, they contribute to long-lasting protection, lubrication, and stabilization of the tear film, addressing both the pathophysiological mechanisms of tear hyperosmolarity and epithelial stress commonly observed in patients receiving chronic antiglaucoma therapy. Clinical investigations have reported significant improvements in Ocular Surface Disease Index (OSDI) scores, tear break-up time, and corneal staining with trehalose–hyaluronate combinations compared with hyaluronate alone [24,25].

The studied formulation also included N-acetyl-aspartyl-glutamate (NAAGA), a medicinal substance known for its soothing and anti-allergic properties. NAAGA has been reported to stabilize mast cells and reduce the release of inflammatory mediators, thereby contributing to a reduction in itching and ocular discomfort [26].

Beyond lubrication, the studied formulation provides additional therapeutic value through components with cytoprotective and anti-inflammatory properties. In particular, N-acetyl-aspartyl-glutamate (NAAGA) has been shown to stabilize mast cells and reduce the release of inflammatory mediators, thereby contributing to the modulation of subclinical inflammation frequently observed in glaucoma patients under chronic topical therapy and in dry eye.

Although NAAGA is not a novel compound, its inclusion in artificial tears represents a potentially beneficial strategy to enhance the anti-inflammatory profile of ocular lubricants [27,28].

The concentration used in the present formulation corresponds to commercially available preparations; however, further studies are warranted to better define dose–response relationships and optimize its clinical use in Ocular Surface Disease, especially in glaucoma. Its inclusion in the ophthalmic solution aims to mitigate subclinical inflammatory responses and sensory irritation associated with chronic exposure to topical glaucoma medications. By modulating inflammatory pathways and decreasing pruritus, NAAGA may further enhance patient comfort and adherence to both artificial tear supplementation and hypotensive therapy.

The magnitude of surface improvement observed in our cohort is clinically relevant. Schirmer test values increased by approximately 4–5 mm and tear break-up time improved by 5 s in both groups, indicating restoration of tear film stability beyond expected measurement variability. These findings are consistent with previous evidence supporting the reparative role of sodium hyaluronate-based artificial tears on epithelial integrity and tear film dynamics [15,16,18,22,23]. Importantly, comparable improvements were observed irrespective of preservative exposure. This suggests that ocular surface compromise in glaucoma therapy may not be exclusively attributable to benzalkonium chloride toxicity but rather to cumulative pharmacologic stress and chronic inflammatory activation [6,7,8]. From a clinical standpoint, this reinforces the concept that surface optimization should not be restricted to preserved regimens but considered broadly in chronically treated patients.

Patient-reported outcomes further strengthen the clinical relevance of these findings. OSDI scores decreased by approximately 19–23 points, exceeding established minimal clinically important differences and reflecting a substantial reduction in symptomatic burden. Artificial tears-related QoL measures improved in parallel, confirming that surface stabilization translates into meaningful patient-perceived benefit. These results align with prior studies demonstrating the strong association between ocular surface instability, treatment dissatisfaction, and reduced QoL in glaucoma populations [10,11,12,13]. Notably, pathology-related QoL scores remained unchanged, supporting the specificity of the intervention’s effect on surface-related discomfort rather than on disease perception itself.

Minor statistically significant VF variations in PSD and VFI were noticed. The better results in terms of VFI may be related to the enhanced optical quality resulting from the use of artificial tears, which may have improved tear film stability and consequently optimized the reliability of VF measurements [29,30,31,32]. Previous studies reported that when artificial tears smooth and rehydrate the ocular surface, they temporarily improve optical quality by smoothing irregularities and improving the refractive surface of the eye. This can lead to better stimulus perception during VF testing.

The VFI is a global parameter derived from automated perimetry that estimates the percentage of visual function remaining. Because VFI is influenced by threshold sensitivity, ocular surface instability from dry eye can artificially reduce it. Therefore, artificial tears may improve tear film quality, enhancing stimulus detection and slightly increasing measured VFI values. These results may justify continuous fluctuations in VF results experienced by glaucomatous patients in the clinical practice.

The clinical implications of these findings extend beyond symptomatic relief. OSD has been consistently associated with reduced adherence and persistence to glaucoma therapy [11,12,13]. By mitigating surface instability and discomfort, adjunctive lubrication may represent a pragmatic strategy to reduce treatment burden and potentially enhance long-term therapeutic continuity. Although adherence was not directly assessed in the present study, the substantial improvements in symptom scores and tear film parameters provide a biologically and behaviorally plausible pathway linking surface optimization to improved treatment tolerability.

This may also explain the improved IOP outcomes observed in both study groups. One possible explanation is that better ocular comfort led to improved adherence, with patients instilling IOP-lowering eye drops more consistently. Another hypothesis is that artificial tears, by reducing ocular surface inflammation and stabilizing the tear film, enhanced corneal permeability and optimized absorption of the IOP-lowering medication [33,34].

In fact, ocular surface inflammation, including dry eye disease, allergic conjunctivitis, or preservative-induced toxicity, disrupts the eye’s natural protective barriers and may reduce the effectiveness of topical medications. Although acute inflammation may transiently increase drug penetration, chronic inflammation generally decreases bioavailability. Inflamed eyes exhibit rapid tear turnover, washing away drops within seconds and limiting contact time. Tear film instability further shortens drug residence on the surface. Damage to the corneal and conjunctival epithelium impairs normal absorption mechanisms. Inflammatory proteins in the tear film may bind medications, reducing tissue penetration. Additionally, faster nasolacrimal drainage increases systemic absorption, lowering intraocular drug concentration and therapeutic efficacy. While acute inflammation may initially increase blood flow and allow more drug to enter, chronic inflammation and a compromised tear film generally lead to poor bioavailability—meaning that less medication reaches the target tissue and more is lost to drainage or systemic absorption [7,35].

Moreover, the increase in Schirmer test values likely reflects indirect restoration of the ocular surface–lacrimal functional unit rather than direct stimulation of tear production. Chronic ocular surface inflammation has been shown to impair lacrimal gland function, and its reduction may restore tear dynamics [36,37].

Furthermore, improved epithelial integrity and tear film stability may enhance tear retention during testing.

This study has several limitations that should be carefully considered when interpreting the results. First, the observational design without randomization may introduce selection bias and limit the ability to establish causal relationships between artificial tear supplementation and the observed improvements. Second, the relatively small sample size reduces statistical power and may limit the generalizability of the findings to the broader glaucoma population. Third, the short follow-up period of one month from one side shows the rapid effects of ocular surface optimization but does not allow the assessment of long-term efficacy, sustainability of benefits, or treatment adherence.

Adherence to artificial tear therapy was assessed by patient self-report at the follow-up visit using standardized questions about daily frequency. No objective adherence measures, such as electronic monitoring or bottle-weight measurement, were employed, which may introduce recall bias and result in overestimation of compliance [38].

Furthermore, tear film stability was evaluated using fluorescein break-up time, which, although widely used in clinical practice, may alter tear film dynamics and provide less accurate measurements compared to non-invasive techniques.

Finally, the study did not include objective biomarkers of ocular surface inflammation or stratification based on disease severity, which could have provided deeper insight into the mechanisms underlying the observed improvements. Future randomized controlled trials with larger cohorts, longer follow-up, objective adherence monitoring, and advanced ocular surface assessment tools are warranted to confirm and extend these findings.

## 5. Conclusions

In conclusion, this prospective study demonstrates that adjunctive artificial tear therapy is associated with significant and clinically meaningful improvements in tear film stability and quality of life in glaucoma patients receiving prostaglandin treatment, regardless of preservative status. These findings support the integration of routine ocular surface assessment and proactive lubrication strategies into comprehensive glaucoma care. Future studies incorporating adherence and persistence endpoints will be essential to determine whether systematic surface optimization may translate into improved long-term therapeutic outcomes.

## Figures and Tables

**Table 1:** Descriptive statistic of considered clinical parameters.

Variable	Group	N	T0 Median (Min–Max)	T1 Median (Min–Max)	*p*-Value *
Visus (decimal)	1	44	0.9 (0.1–1.0)	0.9 (0.3–1.0)	0.002
Visus (decimal)	2	32	0.95 (0.1–1.0)	1.0 (0.3–1.0)	0.001
IOP	1	44	16 (10–20)	15 (10–19)	0.011
IOP	2	32	16 (14–18)	14 (14–18)	0.003
Contrast Sensitivity (%)	1	44	4.4 (2.2–50)	2.2 (1.2–25)	0.001
Contrast Sensitivity (%)	2	32	3.1 (2.2–12.5)	1.9 (1.6–4.4)	0.001
Schirmer (mm)	1	44	5 (2–15)	10 (4–20)	0.001
Schirmer (mm)	2	32	6 (2–12)	10 (6–15)	0.001
BUT (seconds)	1	44	6.5 (1–20)	10 (5–20)	0.001
BUT (seconds)	2	32	5 (3–12)	10 (7–20)	0.001

* Wilcoxon signed-rank test. BUT = break-up time; IOP = intraocular pressure.

**Table 2:** Descriptive statistic of visual field parameters.

Variable	Group	N	T0 Median (Min–Max)	T1 Median (Min–Max)	*p*-Value *
MD	1	44	−4.25 (−23.22–1.72)	−4.70 (−17.19–9.42)	0.188
MD	2	32	−1.03 (−14.46–7.00)	−1.07 (−9.87–13.3)	0.568
PSD	1	44	6.13 (1.23–14.65)	3.54 (1.14–15.38)	0.025
PSD	2	32	2.56 (1.45–10.43)	2.45 (1.50–10.78)	0.548
VFI	1	44	91.0 (28–100)	92.5 (44–100)	0.013
VFI	2	32	96.5 (62–100)	98.0 (78–100)	0.040

* Wilcoxon signed-rank test; MD = Mean Deviation; PSD = Pattern Standard Deviation; VFI = Visual Field Index.

**Table 3:** Descriptive statistic of questionnaires and clinical scales.

Variable	Group	N	T0 Median (Min–Max)	T1 Median (Min–Max)	*p*-Value *
OSDI	1	22	37 (11–65)	17 (0–47)	0.001
OSDI	2	16	39 (18–72)	13.5 (0–39)	0.001
Efron Scale	1	22	3 (0–4)	0 (0–2)	0.001
Efron Scale	2	16	3 (1–4)	0.5 (0–2)	0.001
Therapy-related QoL	1	22	10 (0–50)	7.5 (0–40)	0.038
Therapy-related QoL	2	16	10 (10–30)	10 (0–20)	0.060
Pathology-related QoL	1	22	30 (0–80)	30 (0–80)	0.157
Pathology-related QoL	2	16	30 (15–90)	25 (15–80)	0.063
Artificial tears-related QoL	1	22	0 (0–20)	50 (10–70)	0.001
Artificial tears-related QoL	2	16	7.5 (0–30)	50 (30–80)	0.001

* Wilcoxon signed-rank test; OSDI = Ocular Surface Disease Index; QoL = quality of life.

## Data Availability

The data presented in this study are available on request from the corresponding author due to privacy.
